# Cooperative Friendly Jamming Techniques for Drone-Based Mobile Secure Zone

**DOI:** 10.3390/s22030865

**Published:** 2022-01-24

**Authors:** Ga-Hye Jeon, Ji-Hyun Lee, Yeon-Su Sung, Hyun-Ju Park, You-Jin Lee, Sun-Woo Yun, Il-Gu Lee

**Affiliations:** 1 Department of Future Convergence Technology Engineering, Sungshin Women’s University, Seoul 02844, Korea; 20171988@sungshin.ac.kr (G.-H.J.); nus0205@naver.com (S.-W.Y.); 2 Department of Information & Technology & Data Science & Artificial Intelligence, Sogang University, Seoul 04107, Korea; easyhyun72@gmail.com; 3 Department of Convergence Security Engineering, Sungshin Women’s University, Seoul 02844, Korea; yeonsu1936@gmail.com; 4 Department of Cybersecurity, Korea University, Seoul 02841, Korea; hjpark01@korea.ac.kr; 5 Department of Convergence Security, Sungshin Women’s University, Seoul 02844, Korea; primarily68@gmail.com

**Keywords:** IoT, RF radio communication, Wi-Fi direct, D2D, drone-based mobile secure zone, friendly jamming, mobility

## Abstract

Threats of eavesdropping and information leakages have increased sharply owing to advancements in wireless communication technology. In particular, the Internet of Things (IoT) has become vulnerable to sniffing or jamming attacks because broadcast communication is usually conducted in open-network environments. Although improved security protocols have been proposed to overcome the limitations of wireless-communication technology and to secure safe communication channels, they are difficult to apply to mobile communication networks and IoT because complex hardware is required. Hence, a novel security model with a lighter weight and greater mobility is needed. In this paper, we propose a security model applying cooperative friendly jamming using artificial noise and drone mobility, which are autonomous moving objects, and we demonstrate the prevention of eavesdropping and improved security through simulations and field tests. The Cooperative Friendly Jamming Techniques for Drone-based Mobile Secure Zone (CFJ-DMZ) can set a secure zone in a target area to support a safe wireless mobile communication network through friendly jamming, which can effectively reduce eavesdropping threats. According to the experimental results, the average information leakage rate of the eavesdroppers in CFJ-DMZ-applied scenarios was less than or equal to 3%, an average improvement of 92% over conventional methods.

## 1. Introduction

Wireless communication network technology is evolving to meet the needs of users who want to use high-speed, high-capacity multimedia content without the limitations of location and time. However, cases of information leakage have been continually occurring owing to the fundamental limitations of wireless communication, which is vulnerable to eavesdropping [1]. To solve this problem, protocols and mechanisms have been proposed that improve the security or secure safe communication channels within the time, frequency, and space domains [2]. However, conventional wireless secure communication methods require complex hardware, which reduces the energy efficiency and data transmission performance, limiting the application to highly reliable wireless autonomous moving objects that exchange confidential information [3,4].

Among wireless network technologies, the Internet of Things (IoT) has become an essential element in all industries and everyday life, and accordingly, security vulnerabilities of wireless networks have become a larger issue [5]. Most IoT devices are light in weight with specifications that are insufficient to apply to regular personal computers or mobile devices. The existing communication systems are vulnerable to side-channel attacks, which are attacks that utilize physical information generated from the physical layer. Thus, the security protocol key can be leaked and encryption can be disabled. As they communicate in open network environments, they are easily exposed to security threats such as eavesdropping. This vulnerability can be exploited to easily collect confidential information [6].

To improve the security in IoT environments that are easily exposed to security threats, security architectures should be designed by considering the light weight and mobility of mobile IoT devices [7]. In this paper, we proposed Cooperative Friendly Jamming Techniques for Drone-based Mobile Secure Zone (CFJ-DMZ) to enhance the security in wireless communication environments for drones, which are IoT devices that can move autonomously while exchanging information with other surrounding devices. As eavesdropping is a passive attack that leaves no evidence of attacks, it is impossible to detect eavesdroppers in a wireless communication environment. Therefore, in this paper, a proactive prevention method was proposed to reduce the eavesdropping probability of unspecified potential eavesdroppers. The CFJ-DMZ uses the mobility and artificial interference of autonomous moving objects to form a secure zone, which guarantees safe data communication in the wireless communication environment of mobile IoT and flexibly controls the zone to be protected, effectively mitigating eavesdropping threats.

To evaluate the proposed CFJ-DMZ, we implemented a network simulation model and validated its performance. In the network simulation model, the transmission node moves to near the receiving node, and safe short-range device-to-device (D2D) communication is then conducted in the secure zone formed through a cooperative jammer drone. Here, three drones communicate with each other and create a locally secure zone based on the boundary of the artificial interference signal reaching range to protect the confidentiality of communication between nodes.

The main contributions of this paper are as follows:

- A cooperative friendly jamming technique is proposed to flexibly form a secure zone for confidential communication of lightweight mobile devices.
- The effect of the proposed CFJ-DMZ is analyzed through simulation and proved through field test in the implemented test bed.

The remainder of this paper is organized as follows. Section 2 introduces studies on conventional wireless communication security technologies and major studies on the wireless communication security of autonomous moving objects. Section 3 then describes the security performance criteria of friendly jamming and the friendly jamming model. Section 4 proposes a CFJ-DMZ model, and Section 5 validates the security performance of the proposed model through simulations and field experiments. Finally, Section 6 provides some concluding remarks and describes future research directions.

## 2. Related Work

### 2.1. Introduction to Friendly Jamming

Wyner defined the concept of security capacity in a study on eavesdropping channels and proved that security can be achieved according to the information theory perspective when the quality is poorer in the eavesdropper’s channel than in the legitimate receiver [8]. Security channel capacity (secrecy capacity) is defined as the difference in the channel capacity between a legitimate sender and a legitimate receiver and the channel capacity between a legitimate sender and an eavesdropper. If this value is negative, the security channel capacity is zero, which means that no information can be safely sent. Here, signals and artificial interference can be generated and sent to improve the channel quality of the receiver and deteriorate the channel quality of the eavesdropper. Various studies have been conducted for this, including jamming, beamforming jamming, relay jamming, and friendly jamming techniques for improvements in wireless security. It has recently been proven that if a beam is formed using multiple antennas for jamming signals that can reduce the eavesdropping performance of malicious eavesdroppers, the communication security and reliability between legitimate communicators can be significantly improved. However, beamforming technology that uses multiple antennas or a massive antenna is complex and requires a large power consumption, making it difficult to use in IoT or mobile devices [9,10].

### 2.2. Friendly Jamming Security Model

Various studies are underway on friendly jamming security techniques applying jamming signals for security purposes [11,12,13,14,15,16,17,18]. A friendly jamming technique is a method of emitting friendly artificial interference signals to prevent malicious eavesdroppers from overhearing when the sender communicates with a legitimate receiver. A jammer is used to protect the wireless communication network and send messages with confidentially, allowing legitimate senders to communicate in a secure manner. In friendly jamming security technique research, security metrics are defined to demonstrate the effectiveness and validity of jamming. Friendly jamming security techniques include a method of using anti-jamming that automatically arranges multiple friendly jammers to deliver information safely between moving objects in a mobile communication network [16,17,18].

### 2.3. Friendly Jamming Security Model for Mobility Environment

Research is underway for friendly jamming security models using multiple unmanned aerial vehicles (UAVs) in mobile communication networks. The Friendly UAV Jamming (Fri-UJ) method has shown that the eavesdropping probability decreases as the number of jammers deployed near the protection zone increases because the signal to interference and noise ratio (SINR) of the eavesdropping device drops through the transmitted signals of the jammers [19]. In addition, there is a study that proved its effectiveness by applying Fri-UJ to IoT technology in the medical field [20]. However, using the Fri-UJ, as the number of UAVs increases, the number of jammers increases, thereby increasing the loss cost, and in practice, making it difficult to install an infinite number of jammers. Therefore, research is required to compare, analyze, and propose an effective friendly jamming model in a mobile communication environment based on the size of the secure zone, the number of jammers, and the security efficiency relative to cost.

In addition, research on location optimization considering the mobility of UAVs is being conducted. The location of the drone is important in order to reduce the waiting time and delay of the user or improve the quality of the service provided to users [21]. In [22], the disturbance power intensity and power trajectory path that occur when protecting legitimate nodes located on the ground using friendly jamming signals were studied. At that time, it was calculated by estimating the location of the eavesdropper, but the applicability is low because it is difficult to determine the location of the eavesdropper in the actual situations. Accordingly, in [23], randomness was modeled without identifying the location of the eavesdropper. At that time, in order to maximize the secret ratio of several legitimate receivers, the area was determined using the signal-to-noise ratio (SNR). However, as the confidentiality of the receiver in the security area is not guaranteed equally, some devices have a high potential for eavesdropping. In addition, when the optimal location is determined, only some factors change depending on the situation, so the area is not flexible and the mobility of UAVs cannot be utilized. In the case of battery usage, it is inefficient because jamming signals must be transmitted all the time.

Table 1 shows a summary of prior studies related to friendly jamming. In the table header, “Paper Title” refers to the title of the study, and “Research Topic” refers to the keywords of the study. “Number of Jammers” refers to the number of jammers used in the model: Here, “single” indicates that the model used one jammer, and “multiple” indicates that the model used two or more jammers. In addition, “Main Idea” refers to the model proposed in each paper, and “Limitation” refers to those analysis results that are a limitation of the indicated study. The models in the prior studies are inefficient and inflexible in terms of creating a secure zone for secure wireless communication because they consume large amounts of power and have no mobility. In this study, however, we proposed a model that creates an efficient and flexible secure zone by using only three drones that transmit friendly jamming signals, and we demonstrated the effectiveness of the model through simulations and field experiments.

## 3. Friendly Jamming Technique

Equation (1) shows the information leakage rate (ILR) metrics defined for use as performance evaluation metrics of friendly jamming. The relationship between the bit error rate (BER) and friendly jamming was defined using the ILR. If the BER exceeds 0.5, then the ILR is 0, indicating that it is impossible to extract information because the BIR is high [24,25,26]. By contrast, if the BER is less than or equal to 0.5, then information extraction is possible, and ILR has a value greater than or equal to 0. As the ILR increases, the security decreases, indicating that more information can be extracted.

(1) $$\mathrm{ILR}=0,\;\mathrm{if}\;\mathrm{BER}>0.5\;\mathrm{ILR}=1-\frac{\mathrm{BER}}{0.5},\;\mathrm{if}\;\mathrm{BER}\leq 0.5$$

**Figure 1:**
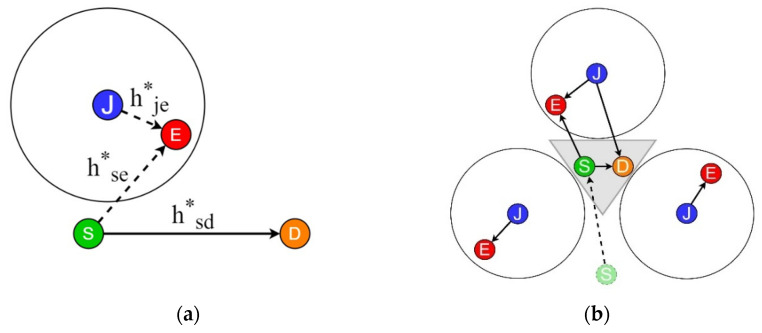
Network configuration for friendly jamming: (**a**) friendly jamming model and (**b**) CFJ-DMZ model.

Figure 1 shows the network configuration for verifying the security performance of friendly jamming. Figure 1a shows a friendly jamming technique model, which uses an open channel to send data ($h_{sd}^{*}$) to the sender node (Source, S) or receiver node (Destination, D) that uses a single antenna. Here, the eavesdropping device (Eve, E) also receives the data ($h_{se}^{*}$). A drone (Jammer, J) located near the source node transmits friendly jamming signals ($h_{je}^{*}$) to form a jamming zone. The eavesdropping quality of the eavesdropping device in the friendly jamming zone deteriorates as an effect of jamming, reducing the possibility of eavesdropping.

## 4. CFJ-DMZ Model

This section introduces the CFJ-DMZ model that forms a secure zone by using the mobility of devices and the friendly jamming method. Three drones transmit jamming signals to the outside of the secure zone. These cooperative jamming signals reduce the eavesdropping probability of eavesdroppers and facilitate secure communication in the secure zone.

Figure 1b shows the CFJ-DMZ model proposed in this paper. The CFJ-DMZ network consists of a source node (S), destination node (D), arbitrary eavesdropper (E), and three friendly jamming drones (J). The drones transmit jamming signals after forming a secure zone, as shown in Figure 1b. The source and destination nodes communicate D2D inside the secure zone. As it is difficult to use a multi-antenna communication interface for low-power lightweight drones, we considered a communication method using a single antenna in this study.

**Figure 2:**
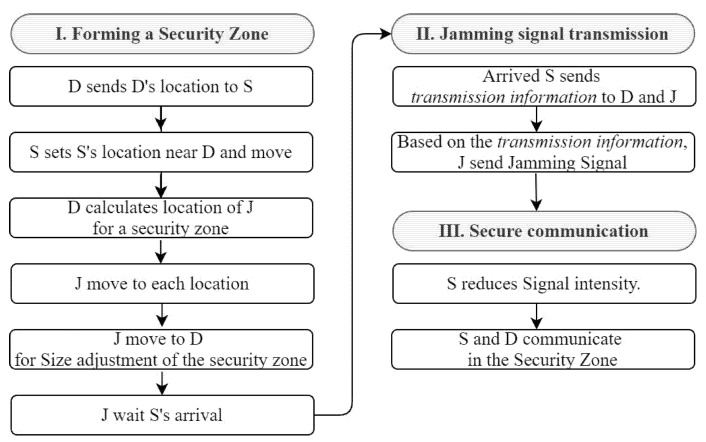
Simulation flow chart.

Figure 2 shows the flowchart of a scenario for the CFJ-DMZ model, which consists of three stages.

In the first stage, three drones are used to form a secure zone. When D2D communication is determined, the receiver (D) delivers its location coordinate information using GPS to the sender (D). In addition, S sends the determined location information to D and moves to the determined location, and D uses the arriving coordinate information of S and the current coordinate information of D to calculate the locations of the drones (J). Moreover, D creates a virtual circle around its position. Here, given that the maximum transmittable distance according to the strength of the transmitted jamming signals of J is x, the radius of the circle is as shown in Equation (2):

(2) $$\mathrm{Radius}=\frac{2}{3}\sqrt{3}x$$

Here, D selects three arbitrary points to make an equilateral triangle on the virtual circle created by centering on the position of D and sending the selected location to J. Each J moves to the received location. The J that arrives at each location moves to D until their respective jamming signals are no longer caught while transmitting to adjust the size of the secure zone. After the secure zone is created, J stops sending the jamming signals and waits until S arrives at the final target coordinates.

The second stage is the jamming signal transmission stage. After arriving at the final target coordinates, S sends the jamming signal transmission start time and the jamming signal transmission maintenance time to both D and J. The three units of J transmit jamming signals simultaneously based on the time information, and S and D can send secure data during the jamming signal transmission maintenance time. As such, J sends jamming signals only when communication is made according to the data transmission time. If jamming signals are continuously transmitted irrespective of the data transmission, the security inside the secure zone improves. However, continuous jamming signals interfere with the communication of other nearby transmitting and receiving objects [27]. Furthermore, it is inefficient to transmit jamming signals continuously in terms of energy. In the CFJ-DMZ, J therefore sends jamming signals only when S and D are communicating to minimize the effects of such signals on other nearby sender and receiver nodes and to use their batteries efficiently. In addition, it is assumed that the secure zone is formed only when the legitimate nodes exchange confidential information with the help of surrounding UAVs used for other purposes. Therefore, the cost of friendly jamming drones for CFJ-DMZ was not considered in this paper. On the other hand, very small control logic can be added to friendly jamming drones and the ground user’s hardware to implement the proposed method, but it is assumed that the added hardware cost is trivial.

Finally, S and D communicate inside the secure zone formed by the jamming-signal transmission of J. Here, because the distance between S and D is close, the transmission signal strength of S is reduced. The three units of J transmit cooperative jamming signals. As a result, the probability of success of the eavesdroppers decreases, improving the security of D2D communication in the secure zone.

In this study, we conducted simulations and field experiments to prove the security of the CFJ-DMZ model. Octave was used for the simulations, and Raspberry Pi 3 was used in the field experiments. Section 5 discusses the security verification process and results. The experiments were focused on proving the security improvement inside the secure zone. Therefore, as a part of the process under the CFJ-DMZ scenario, we assumed that the source node has moved and that the friendly jamming drones have completed moving to form a secure zone.

## 5. Experiment

### 5.1. Simulation

#### 5.1.1. Effect of Friendly Jamming

**Figure 3:**
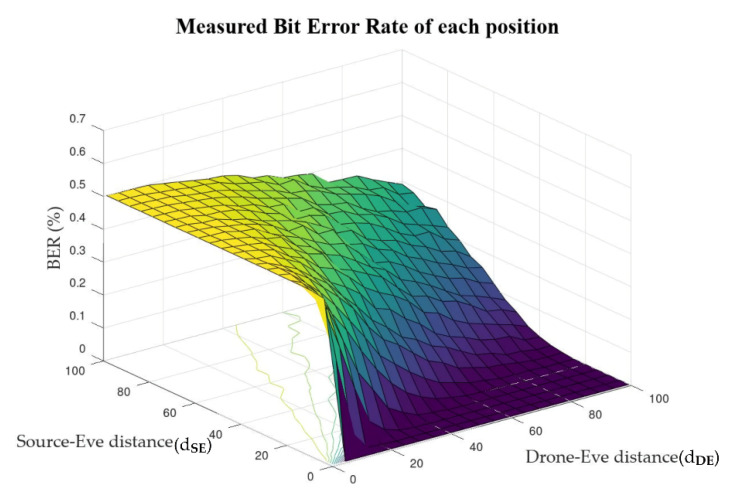
BER based on distance of Source-Eve and Drone-Eve.

The CFJ-DMZ model proposed in this paper uses three drones as friendly jammers to protect the confidentiality of D2D communication. At this time, the three drones are theoretically the smallest number to make a two-dimensional space, and they protect the communication of the two legitimate nodes by forming a cost-performance effective secure zone. The jamming effect on the eavesdropper is affected by the distance between the source node and the eavesdropper (Source − Eve distance, d_SE_) and the distance between the jamming drones and the eavesdropper (Drone − Eve distance, d_DE_). Figure 3 shows the BER of the eavesdropper according to changes in d_SE_ and d_DE_ measured using the simulation. The BER of the eavesdropper was measured by changing each distance from 1 to 100 m. As a result, we found that when d_DE_ is shorter than d_SE_, the BER of the eavesdropper increases, reducing the communication quality of the eavesdropper.

#### 5.1.2. Evaluation Environments

The evaluation simulator was implemented using Octave v.6.1. The simulation was conducted in a PC environment with the Windows 10 operating system, 8 GB of RAM, and an Intel i5-7200U CPU. In the simulation, a free space of 200 m × 200 m was formed, and the location of nodes was randomly arranged to prove the effectiveness of the CFJ-DMZ. The experimental environment was set as a free space that did not take into account the influence of air or other radio waves. In addition, the maximum transmission power of the transmitting node, receiving node, and friendly jamming drone in the simulation was 24 dBm each.

**Figure 4:**
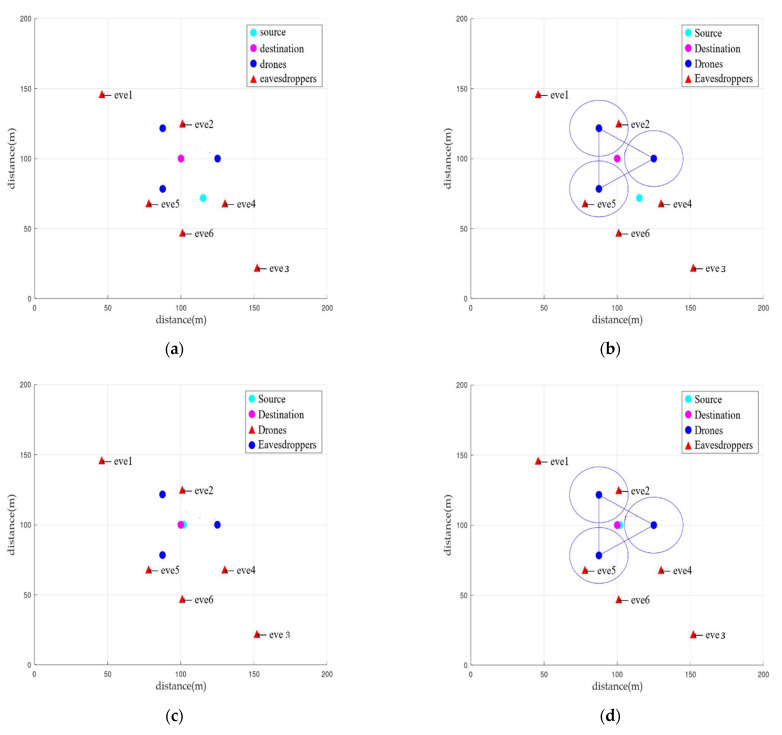
Network configuration for CFJ-DMZ simulation: (**a**) None mobility and None friendly jamming, (**b**) None mobility and Friendly jamming, (**c**) Mobility and None friendly jamming, and proposed scheme (**d**) Mobility and Friendly jamming.

We configured four experimental settings to measure the leaked amount of information according to the mobility of the device and the friendly jamming technique. Figure 4 shows each experimental configuration.

After measuring the locations of the source node (S) and destination node (D), the locations of three drones (J) were calculated. The blue circles in Figure 4b,d represent the range in the cooperative jamming signals. A total of six eavesdropping nodes (eve1–6) were created, and the eavesdropping nodes (E) were located at arbitrary coordinates. The locations of the eavesdroppers were the same in all experiments for an effective comparison between the experiments. Table 2 shows a summary of the location of each node. In the simulation experiments, the distance from the source node to the destination source was set to 48.413 m in Figure 4a,b and to 2 m in Figure 4c,d.

The test cases of the experimental settings in Figure 4 can be summarized as shown in Table 3. In the table, “mobility” refers to with or without movement of the source node. If the mobility is O, it indicates a case in which the source node sends information after moving to near the destination node. If the mobility is X, it indicates a case in which the source node sends information without moving to near the destination node. When the source node has mobility, and the distance between the source and destination nodes is close, the transmission signal strength of the source node is reduced. “Friendly jamming” refers to whether the friendly jamming technique of the drone is used. If friendly jamming is O, it indicates a case in which the drones transmit cooperative jamming signals when sending information. If it is X, it indicates a case in which friendly jamming signals are not transmitted. “Source-Destination distance” refers to the distance between the source and destination nodes. The CFJ-DMZ model corresponds to Figure 4d in which both mobility and friendly jamming occur.

The design process of the simulation is as follows. First, it is assumed that there is a channel $h_{sd}^{*}$ in the form of a complex conjugate between the source and destination nodes. It is also assumed that $P_{s}$ at the transmitter and the receiver, and the maximum transmission power of $P_{j}$ of the friendly jamming drones, are both 24 (dBm). Under these assumptions, the following equations are defined to measure the BER at each object.

First, the signal that the destination node receives from the source node in Equation (3) can be expressed based on the distance between the source and destination nodes ($h_{sd}^{*}$), the distance between the jammer drones and the destination node ($h_{jd}^{*}$), the maximum transmission power of the receiver ($P_{s}$), the maximum transmission power of the jammer drones ($P_{j}$), and the noise ($n_{d}$) [28]:

(3) $$y_{e}=G\sqrt{P_{s}}h_{sd}^{*}s+\sqrt{P_{j}}\;h_{jd}^{*}q+n_{d}$$

where the channel coefficient h is shown by Equation (4). In addition, d is the distance between the two communication nodes, e is a uniformly distributed random number a+bi, and c is the path loss exponent.

(4) $$h={\left(d\right)}^{\frac{-c}{2}}e$$

The amplification scale vector G can be shown through Equation (5), where N refers to the Gaussian noise.

(5) $$G=\frac{1}{\sqrt{P_{s}{\left|h_{sd}^{*}\right|}^{2}+N}}$$

Finally, the signal received by the eavesdropper in Equation (6) can be represented by the distance between the source node and the eavesdropper ($h_{se}^{*}$), the distance between the jammer drones and the eavesdropper ($h_{je}^{*}$), the maximum transmission power of the receiver ($P_{s}$), the maximum transmission power of the jammer drones ($P_{j}$), and the noise ($n_{d}$).

(6) $$y_{e}=G\sqrt{P_{s}}h_{se}^{*}+\sqrt{P_{j}}h_{je}^{*}q+n_{e}$$

For the BER of the eavesdropper node in each experiment, we used an average of 1000 times, as shown in Equation (7). In Figure 4a,c, where there is no jamming signal, $G\sqrt{P_{j}}h_{je}^{*}*JamSymbols$ is calculated as zero.

(7) $$y_{e}=G\sqrt{P_{s}}h_{se}^{*}*\;TrsutSymbols+G\sqrt{P_{j}}h_{je}^{*}*JamSymbols$$

Table 4 shows the definitions of the parameters used in the simulation pseudo-code, and Algorithm 1 shows the simulation pseudo-code itself. The detailed operating principle of Algorithm 1 is as follows.

In line 1–5, the distance between the jamming drones and eves is measured, and the measured distance is used for the influence of jammer on eve. Line 6 refers to the number of simulation repetitions, and the average BER is evaluated by repeating 1000 times. The c of line 7 is the path loss index and is used to evaluate the channel coefficient. Line 8–24 is a function of calculating the average BER of the eavesdropper and is repeatedly performed by a value specified in line 6. This function receives the size of data to be transmitted, the path loss index, the maximum source power of jammer, and the distance between source node and eavesdropper, source node and destination node, and jammer and receiver as parameters. The e of line 10 is a random number in the form of a complex number and is used to evaluate the channel coefficient. In line 11–13, channel coefficients are calculated using c and e. The G of line 14 is a scaling coefficient amplified according to the distance between the source node and the destination node. In line 15–16, 0 and 1 are randomly generated as the size of the data to be transmitted and then mapped in a complex number form. In line 17–18, data to be used as jamming signals are randomly generated and then mapped in complex number form. Line 19 refers to a signal received by the eavesdropper, and line 20 refers to the bit formed as signals. In line 21, the BER is calculated using the bits sent by the source node and the bits received by eve. In line 22, the average BER is evaluated.

**Algorithm 1.** Pseudo-code for BER measurement of eve.1:

$Drone_{1}toEveDistatnce\;\leftarrow Distance\;between\;Drone_{1}\;and\;Eve$

2:

$Drone_{2}toEveDistatnce\;\leftarrow Distance\;between\;Drone_{2}\;and\;Eve$

3:

$Drone_{3}toEveDistatnce\;\leftarrow Distance\;between\;Drone_{3}\;and\;Eve$

4:

CalculateJEDistance ←Calculate the distance of the cloest drone from eve

5:

jeDistance←The distance between one jammer and the eavesdropper affected by the jammers

6:

maxLoop ←1000

7:

c ←path loss exponent

8: $procedure\;MeasureEveBER\;(N_{bits},\;c,\;seDistance,\;sdDistance,\;jeDistance,\;P_{t},\;P_{j}$)9:  for 1 to maxLoop
10:   $e\;\leftarrow randomComplexNumber\left(N_{bits}\right)$
11:   $h_{se}\;\leftarrow seDistance^{-\frac{c}{2}}*e$
12:   $h_{sd}\;\leftarrow sdDistance^{-\frac{c}{2}}*e$
13:   $h_{je}\;\leftarrow jeDistance^{-\frac{c}{2}}$
14:   $G\;\leftarrow \;\frac{1}{\sqrt{\left(p_{t{\left|h_{sd}\right|}^{2}}\right)}}\;$
15:   $SignalBits\;\leftarrow Generate\;randomly\;Signal\;Bits\;of\;N_{bits}\;at\;0,1$
16:   SignalSymbols ←Mapping SignalBits to SignalSymbol in the form of complex number
17:   $JamBits\;\leftarrow Generate\;randomly\;JAmming\;Symbol\;of\;N_{bits}\;at\;0,1$
18:   JamSymbols ←Mapping JamBits to JamSymbol in the form of complex number
19   $eveRecieveSymbol\;\leftarrow G*\sqrt{P_{t}}*h_{se}*SignalSymbols+\sqrt{P_{j}}*h_{je}*JamSymbols$
20:   eveRecieveDemappedBits ←Demapping eveRecieve Symbol to Bits
21:   MeasureBER ←Sum(SignalBits ≠eveRecieveDemapped Bits)Nbits, Calculate BER with comparision between Signal Bits and eveRecieve Demapped Bits22:   average BER ←Take average for the BER results of each loop so the BER has minimized the bias
23:  end for
24: end procedure


#### 5.1.3. Results of Simulation

**Figure 5:**
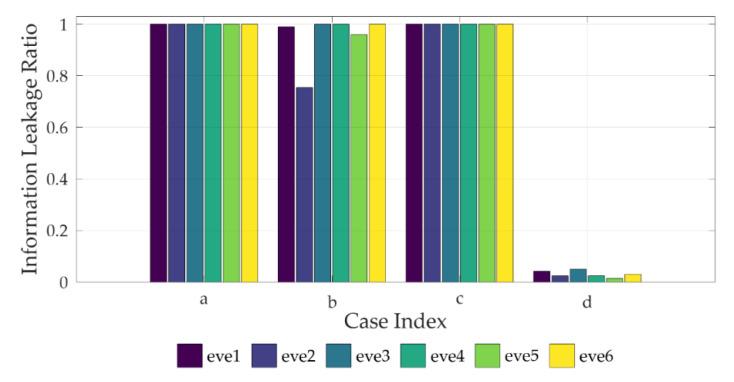
Simulation results: conventional schemes (**a**–**c**) and proposed scheme (**d**).

The source node transmitted 100,000 SignalSymbol data, and the BER was measured for the destination node and the eavesdroppers. The average BER was obtained by repeating the simulation 1000 times. In every case, the average BER of the destination node was 0, which means that all data sent by the source node were received. Figure 5 shows a graph that applies the average BER results of the eavesdroppers to the ILR metrics defined in Section 3.

In Figure 4a, the ILR of every eavesdropper is 1. Therefore, if the devices have no mobility and the friendly-jamming technique is not used, the eavesdropping probability of the eavesdroppers is high.

In Figure 4b, the characteristics of Wang’s Friendly UAV Jamming model (Fri-UJ) [19] are included. Fri-UJ reduces the eavesdropping probability by using multiple UAVs as jammers to transmit friendly jamming signals in a mobile communication network environment. However, unlike CFJ-DMZ, the mobility of the source node is not considered. At this time, the ILR of eve2 and eve5, which are within the cooperative jamming signal range of the drones, is 0.754 and 0.960, respectively. The ILR of the eavesdroppers outside the cooperative jamming signal range of the drones is 1. Therefore, if the friendly jamming technique is used without the mobility of the devices, the eavesdropping probability of eavesdroppers is high.

In Figure 4c, the ILR of every eavesdropper is 1. As the distance between the source and destination nodes is close, the signal strength of the source node is reduced. However, it does not have a significant impact on the communication quality of the eavesdroppers, because the simulation environment is a free space. Therefore, if the devices have mobility but the friendly jamming technique is not used, the eavesdropping probability of the eavesdroppers is high.

In Figure 4d, which is the case of the CFJ-DMZ model, the ILR of eve2 and eve5, which are within the cooperative jamming signal range of the drones, is 0.024 and 0.014, respectively. Furthermore, the ILR of the eavesdroppers outside the cooperative jamming signal range of the drones is close to zero, and the average ILR of all eavesdroppers is 0.03. Therefore, when the mobility of the devices and the friendly-jamming technique are used together, the communication quality of the eavesdropper decreases. Compared to Figure 4b that includes the characteristics of Fri-UJ, the average ILR is reduced by 92%, and compared to Figure 4a,c, the average ILR is reduced by 97%, facilitating secure communication in the secure zone.

### 5.2. Field Experiment

#### 5.2.1. Effect of Friendly Jamming

In this section, we conducted experiments using Raspberry Pi. The friendly jamming drones and the eavesdroppers were also implemented using Raspberry Pi. In the communication between Raspberry Pi devices, packets were sent using the D2D communication method. The source node became the AP using the host mode, and the destination node was connected to the AP of the source node. The maximum transmission power of each node was 24 dBm. Jamming signals were generated using the Ping of Death method.

**Figure 6:**
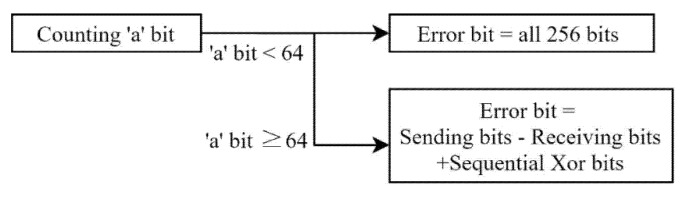
Synchronization with preamble bit.

For the data, a string “1” consisting of a total of 256 bits was used, and as the preamble bits for synchronization, a string “a” consisting of 128 bits was used. Figure 6 shows how the preamble bits were processed. If “a” with less than 64 bits is received, it corresponds to a case in which the ILR is less than 0.5. Therefore, the data received through the corresponding packet are all processed as an unanalyzable state. If “a” with 64 or more bits is received, the number of error bits is obtained after removing the preamble data. The number of error bits is calculated by adding the number of lost bits and the number of unmatched bits. The number of lost bits is calculated by subtracting the number of receiving bits from the number of sending bits, and the number of unmatched bits is calculated by counting 1 after the sequential Xor. The BER of the destination node and the eavesdroppers is obtained using the error bits.

First, the friendly jamming model of Figure 1a was implemented to verify the effectiveness of the jamming signals. One jamming drone node was deployed based on the locations of the source and destination nodes.

The transmitted data of the source node, and the BER of both the destination node and the eavesdroppers, were measured. Table 5 shows the average BER obtained by repeatedly applying the model of Figure 1a 1000 times. The average BER of the destination node is zero. In other words, the destination node can communicate normally with the source node because it is unaffected by the jamming signals. The average BER of the eavesdroppers located around the drone nodes (eve3, eve4, and eve5) is 0.975, 0.992, and 0.938, respectively, showing a result close to 1. This experimental result shows that the legitimate destination node can safely deliver information while reducing the amount of information leaked to the surrounding eavesdroppers by friendly jamming. Furthermore, the average BER of the eavesdroppers that are not located around the drones (eve1, eve2, and eve6) is 0.389, 0.012, and 0.562, respectively, demonstrating relatively low values. Therefore, if the friendly jamming technique is used, the eavesdropping probability of the eavesdroppers decreases, improving the communication security.

#### 5.2.2. Experimental Settings

The field experiment was executed using Raspberry Pi 3 Model B+. The experiment was conducted in an empty lot of 50 m × 50 m with a Quad-core 64-bit ARMv8 CPU and 1 GB of RAM. At this time, the empty lot was used to minimize the influence of other radio waves. The maximum transmission power of Raspberry Pi is 24 dBm, so the maximum transmission power of the source node, receiving node, and friendly jamming drone used in the experiment is 24 dBm each. The field experiments were conducted under the same environmental configuration as used in the simulations. That is, the devices were placed according to the experimental settings of Figure 4. Similar to the simulations, to achieve an effective comparison between the experiments, the locations of the eavesdropper were the same in every experiment. Furthermore, considering the signal ranges of the source node and the jamming nodes, we placed the eavesdroppers at the locations where the effects of the signals received according to the experimental environment were the same as those received by the eavesdroppers during the simulations.

#### 5.2.3. Experiment Results

**Figure 7:**
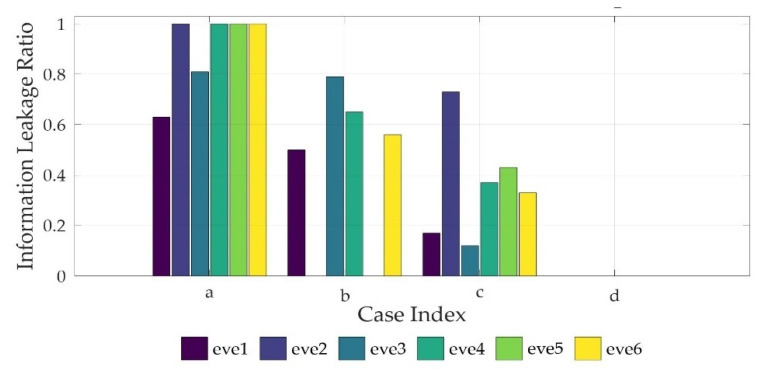
Field experiment results; conventional schemes (**a**–**c**) and proposed scheme (**d**).

The BERs of the destination node and the eavesdroppers, respectively, were measured when the source node sent the data. The average BER was obtained by measuring the BER 1000 times repeatedly. In every case, the average BER of the destination node was 0, which means that the data sent by the source node were all received. Figure 7 shows a graph applying the average BER results of the eavesdroppers to the ILR metrics defined in Section 3.

In Figure 4a, the ILR of the eavesdroppers, except for eve1 and eve3, is 1. The ILR of eve1 and eve3 is 0.639 and 0.819, respectively. Therefore, if the devices have no mobility and the friendly jamming technique is not used, the eavesdropping probability of the eavesdroppers is high. Furthermore, it was found that, as the distance between the eavesdropper and the source node increases, the eavesdropping probability of the eavesdropper decreases.

In Figure 4b, the ILR of every eavesdropper decreases because the cooperative jamming signals affect the communication quality of the eavesdroppers. The ILR of both eve2 and eve5, which are within the cooperative jamming signal range of the drones, is zero. However, the ILR of the eavesdroppers outside the cooperative jamming signal range of the drones is greater than or equal to 0.5. Therefore, if the friendly jamming technique is used without the mobility of the devices, the eavesdropping probability of the eavesdroppers outside the cooperative jamming signal range is high.

In Figure 4c, the transmission signal strength is reduced because the distance between the source and destination nodes is close. As a result, the ILR of eve1 and eve3 is 0.180 and 0.121, respectively. However, the ILR of eve2, eve4, eve5, and eve6, which are relatively close to the source node, is 0.732, 0.380, 0.433, and 0.338, respectively. Although the ILR of every eavesdropper decreases compared to that of Figure 4a, the ILR of some eavesdroppers is high. Therefore, if the devices have mobility but the friendly jamming technique is not used, the eavesdropping probability of the eavesdroppers is high.

In Figure 4d, which is the case of the CFJ-DMZ model, the ILR of every eavesdropper is zero. In other words, when the mobility of the devices and the friendly jamming technique are used together, the communication quality of the eavesdroppers decreases, reducing the eavesdropping probability. Therefore, secure communication is facilitated in the secure zone.

### 5.3. Evaluation

The results obtained from the simulations and the field experiments are as follows. Based on the ILR of eve1 and eve3 in Field Experiment Figure 4a, it was found that as the distance between the source node and the eavesdropper increases, the communication quality of the eavesdropper deteriorates.

Furthermore, based on the ILR of eve5 and eve6 in Figure 4b, it was found that as the eavesdropper reaches closer to the friendly jamming drones, the communication quality of the eavesdropper deteriorates.

A comparison of the average ILR of the eavesdroppers between Figure 4a–d shows the effect of the device mobility. It can be seen that when the source node moves to the receiving node to reduce the distance between the two devices and decreases the transmission signal strength, the communication quality of the eavesdroppers deteriorates.

In Figure 4d, where the mobility of the devices and the friendly jamming technique are used together, the average ILR of the eavesdroppers is 0.03 in the simulation results and zero in the field experiment results. This means that the CFJ-DMZ model can reduce the communication quality of eavesdroppers to reduce the eavesdropping probability in the zone where the possibility of eavesdropping is high. Therefore, the communication security in the secure zone improves.

## 6. Conclusions

In this paper, we proposed the CFJ-DMZ method, which improves the security of wireless communication by using the mobility of mobile IoT devices and jamming signals, and we verified its effectiveness in drone communication environments. The drones conducting the cooperative friendly jamming move to locations where a secure D2D communication will be performed and transmit jamming signals to form a secure zone. The formed secure zone can effectively prevent eavesdropping and is flexible because the location and size can be easily changed. Furthermore, because jamming signals are transmitted only when the data transmitter and receiver communicate, the effect of the jamming signals on other source and destination nodes is minimized, and the batteries are efficiently used. Through the CFJ-DMZ model-applied simulations and fields tests, the BER of the eavesdropping devices was measured, which confirmed that the receiving performance of the eavesdroppers deteriorated, reducing the normal packet reception rate. Furthermore, we defined the ILR as a metric for a security performance evaluation and confirmed experimentally that the information leakage decreased with the proposed scheme.

By integrating it with IoT networking environments across future social systems, including logistics, delivery, and unmanned moving objects, the proposed CFJ-DMZ method can be used not only for military drone communications but also as a model that can actually be commercialized. As a limitation of this study, the effects of jamming were examined in two-dimensional planes. To consider the intrinsic emission characteristics of RF, the effectiveness of the CFJ-DMZ method should also be verified in three dimensions. Furthermore, low latency is important for the proposed method to be applied to real-time systems, not only for delay-tolerant applications. However, in this study, time complexity could not be analyzed, and the study was conducted from the perspective of an information leakage ratio to verify whether confidential communication is possible using the proposed method. Therefore, experiments and verifications are required for various environments, including a case in which an eavesdropper enters the secure zone formed by the drones. In follow-up studies, we will make improvements on this limitation by applying actual environmental parameters to mathematical models and simulation models for assessment of time and resource complexity. Based on these studies, we expect to design a more advanced security architecture and increase the level of security in confidential and complex zones.

## Figures and Tables

**Table 1:** Comparison of prior studies on friendly jamming.

Paper Title	Research Topic	Number of Jammers	Main Idea	Limitation
UAV-enabled friendly jamming scheme to secure industrial Internet of Things [19]	Eavesdropping, Internet of Things, unmanned aerial vehicles	Multiple	Unmanned aerial vehicles (UAV)-enabled friendly jamming scheme	Need to study the optimal number of jammers
A jamming approach to enhance enterprise Wi-Fi secrecy through spatial access control [16]	Eavesdropping, Wi-Fi networks	Multiple	Defensive jamming approach in Wi-Fi networks secured by the WPA2 enterprise mode	Need to study friendly jamming techniques in a mobile environment
Achieving physical layer security with massive MIMO beamforming [17]	Antenna arrays, beamforming, cylindrical arrays, massive MIMO	Single	Beamforming with large cylindrical antenna arrays	Need to study appropriate node placement
Friendly jammer against an adaptive eavesdropper in a relay-aided network [14]	Relay-aided single-input single-output network, adaptive eavesdropping, outage probability	Single	Simulations for improvement in the secrecy capacity and SOP performances owing to the presence of friendly jamming	Need to study improved security for confident communication of nodes that want protection, and optimal number of jammers
Friendly jamming for wireless secrecy [12]	Cooperative jamming, jamming coverage, jamming efficiency, eavesdropping	Single	Cooperative/friendly jamming on the secrecy outage probability of a quasi-static wiretap fading channel	Need to study friendly jamming techniques in a mobile environment, and the optimal number of jammers

**Table 2:** Location of each node in simulation.

Node	Source in Figure 4a,b	Source in Figure 4c,d	Destiantion	eve1	eve2	eve3	eve4	eve5	eve6
Location	(127.42, 60.1)	(102, 100)	(100, 100)	(23, 146)	(101, 125)	(155, 22)	(149, 89)	(73,71)	(101, 47)

**Table 3:** Environment setting of Figure 4.

Case	Figure 4a	Figure 4b	Figure 4c	Figure 4d
Mobility	X	X	O	O
friendly jamming	X	O	X	O
Source-Destination distance (m)	48.413	48.413	2	2

**Table 4:** Defined parameters for pseudo-code.

Notation	Remark
c	Channel coefficient for free-space path loss
e	Randomized complex number
$h_{se}$	Channel coefficient for free-space path loss of distance between source and eve
$h_{sd}$	Channel coefficient for free-space path loss of distance between source and destination
$h_{je}$	Channel coefficient for free-space path loss of distance between jammer and eve
G	Scaling factor of amplification based on the distance source and destination

**Table 5:** Results of friendly jamming (field experiment).

Node	Destination Node	eve1	eve2	eve3	eve4	eve5	eve6
BER metric	0	0.389	0.012	0.975	0.992	0.938	0.562

## Data Availability

Not applicable.

## Glossary/Nomenclature/Abbreviations

The table summarizes the notation used in the paper.

Nation	Meaning
CFJ-DMZ	Cooperative Friendly Jamming Techniques for Drone-based Mobile Secure Zone
IoT	Internet of Things
D2D	Device to Device
UAV	Unmanned Aerial Vehicle
Fri-UJ	Friendly UAV Jamming
SINR	Signal to Interference and Noise Ratio
ILR	Information Leakage Rate
BER	Bit Error Rate
S	Source
D	Destination
E	Eve
J	Jammer
d_SE_	Source-Eve distance
d_DE_	Drone-Eve distance
